# IFNβ enhances mesenchymal stromal (Stem) cells immunomodulatory function through STAT1-3 activation and mTOR-associated promotion of glucose metabolism

**DOI:** 10.1038/s41419-019-1336-4

**Published:** 2019-01-28

**Authors:** Tiziana Vigo, Claudia La Rocca, Deriggio Faicchia, Claudio Procaccini, Maddalena Ruggieri, Marco Salvetti, Diego Centonze, Giuseppe Matarese, Antonio Uccelli, on behalf of the MSRUN Network

**Affiliations:** 1 IRCCS Ospedale Policlinico San Martino, Genoa, Italy; 2 Laboratorio di Immunologia, Istituto di Endocrinologia e Oncologia Sperimentale, Consiglio Nazionale delle Ricerche (IEOS-CNR), Naples, Italy; 3 IRCSS Fondazione Santa Lucia, Rome, Italy; 4 Department of Basic Medical Science, Neuroscience and Sense Organs, University of Bari, Bari, Italy; 5 Centre for Experimental Neurological Therapies (CENTERS), Sapienza University, Rome, Italy; 6 IRCCS Istituto Neurologico Mediterraneo (INM) Neuromed, Pozzilli, Italy; 7 Laboratory of Synaptic Immunopathology, Tor Vergata University, Rome, Italy; 8 Dipartimento di Medicina Molecolare e Biotecnologie Mediche, Università degli Studi di Napoli “Federico II”, Naples, Italy; 9 Department of Neurosciences, Rehabilitation, Ophthalmology, Genetics, Maternal and Child Health Unit and Center of Excellence for Biomedical Research, University of Genoa, Genoa, Italy

## Abstract

Administration of mesenchymal stem cells (MSC) ameliorate experimental autoimmune encephalomyelitis (EAE), a mouse model of multiple sclerosis (MS), at both clinical and neuropathological levels. The therapeutic properties of MSC in EAE are mainly mediated by the modulation of pathogenic immune response, but other neurotropic effects, including decreased demyelination and axonal loss as well as promotion of tissue repair, play also a role. Properly controlled phase II clinical trials to explore the potential of MSC transplantation as a treatment for MS are underway. Interferon beta (IFNβ) is an approved treatment for relapsing-remitting and secondary progressive MS. Here, we explored the possibility that IFNβ might influence the therapeutic potential of MSC, in view of possible synergistic effects as add-on therapy. IFNβ enhanced the immunomodulatory functions of MSC and induced the expression of secretory leukocyte protease inhibitor (*Slpi)* and hepatocyte growth factor (*Hgf)*, two soluble mediators involved in immune and regenerative functions of MSC. At molecular level, IFNβ induced a rapid and transient phosphorylation of STAT1 and STAT3, the transcription factors responsible for *Slpi* and *Hgf* induction. Concomitantly, IFNβ dynamically affected the activity of mTOR, a key checkpoint in the control of metabolic pathways. Indeed, the impairment of mTOR activity observed early upon exposure to IFNβ, was followed by a long-lasting induction of mTOR signaling, that was associated with an increased glycolytic capacity in MSC. When induced to switch their energetic metabolism towards glycolysis, MSC showed an improved ability to control T-cell proliferation. These results suggest that modifications of MSC energetic metabolism induced by IFNβ may contribute to promote MSC immunomodulatory function and support a role for metabolic pathways in the therapeutic function of MSC. Altogether, these findings support the idea of a combined treatment for MS, in which the immunomodulatory and possibly regenerative activity of MSC could be enhanced by the administration of IFNβ.

## Introduction

Mesenchymal Stromal (Stem) Cells (MSC) are a heterogeneous subset of stromal progenitors of mesodermal lineage that have been isolated from almost every tissue, mainly the bone marrow and adipose tissue. MSC are defined on the basis of their capability to grow as adherent cells on plastic, to display a fibroblast-like morphology, to form colonies in vitro supporting hematopoiesis, to differentiate into cells of the mesodermal lineage and express stromal while lacking hematopoietic markers^1^. Several studies showed that MSC possess immunomodulatory properties exerted on cells populations of both adaptive and innate immunity^2^ and these features, together with their reported ability to protect neural cells from death and foster neural repair, account for their proposed therapeutic effect on multiple sclerosis (MS) and other neurological diseases^3^. Intravenous infusion of MSC improved the clinical course of EAE, inducing immune tolerance, reducing inflammation decreasing demyelination and promoting tissue repair^4–9^. The mechanisms through which MSC exert their therapeutic function are heterogenous and probably pleiotropic. It is generally accepted that MSC immunomodulation is strongly influenced by cytokines in the inflammatory environment, particularly by IFN gamma (IFNγ)^10,11^ and that their therapeutic effect is mediated by paracrine mechanisms through the release of soluble factors^2^. Particularly, intravenous injection of conditioned medium containing MSC-derived hepatocyte growth factor (HGF) promoted remyelination in vitro and tissue repair in vivo^12^. Autologous MSC have been safely administrated in a limited number of patients with MS^13^, and ongoing controlled clinical studies to explore the potential of MSC transplantation as a treatment for MS have been launched (Clinical Trial NCT01854957).

Interferon beta (IFNβ) is an approved treatment for relapsing-remitting^14^ and secondary progressive multiple sclerosis (MS). Interferons are a family of cytokines secreted by various cell types of the innate and adaptive immune systems as well as by other tissues. One major pathway in IFNβ signaling involves activation of Signal Transducer and Activator of Transcription (STAT) proteins and formation of complexes that translocate to the nucleus and bind to specific elements to regulate gene transcription^15^. Efficacy of IFNβ for the treatment of MS is believed to be due to modulation of immune responses^16^. Among its functions, in MS subjects IFNβ modulates dendritic cells^17^, T and B lymphocytes^18^, as well as regulatory NK cells and T regulatory cells^19^.

While the potent immunomodulatory effect of IFNβ on cells of the immune system has been extensively studied, little is known on the interaction between IFNβ and MSC. We recently demonstrated that the pro-immunomodulatory effect of IFNγ on MSC is mediated by the phosphorylation of STAT1 and STAT3 and by the inhibition of mammalian target of rapamycin (mTOR) activity^11^. Thus, we sought to address the effect of IFNβ on MSC immunomodulatory functions keeping in mind that, based on the possibility of a synergic effect, these two treatments could be effectively associated to treat MS.

Our data showed that IFNβ promoted the ability of MSC to control T-cell proliferation and enhanced the gene expression of *Hgf* and of secretory leukocyte protease inhibitor (*Slpi)*, an important regulator of innate and adaptive immunity and a component of tissue regenerative programs^20^. At molecular level IFNβ induced a rapid activation of STAT1 and STAT3 in MSC, concomitant with an early inhibition followed, at 24 h, by a strong re-activation of mTOR pathway. The oscillatory switch of mTOR signaling induced by IFNβ was associated with an increased glycolytic capacity of MSC, that improved their ability to control T-cell proliferation.

## Materials and methods

### MSC culture and treatments

MSC isolated from mouse bone marrow were cultured in Mesencult medium as described [8]. MSC were treated with 1500 UI/ml of IFNβ (Rebif ® 44, Merk Serono) for 1 h in serum-free RPMI (Gibco).

### Gene silencing

siRNA transfection was carried out using Lipofectamine 2000 in serum free RPMI for 24 h in the presence of specific siRNA (20 pmol). STAT1:(gauugaccuggagaccaccucucuu/aagagagguggucuccaggucaauc)

SLPI: (caagugcugugaggguaua(dt)(dt)/uauacccucacagcacuug(dt)(dt)

As negative control, MSC were transfected with AllStar siRNA (Qiagen) (CTRL-KD) according to manufacturer’s instructions. The efficacy of siRNA knock-down was tested by real time PCR.

### Collection of MSC-conditioned medium

Confluent MSC were primed with IFNβ for 1 h in serum-free RPMI. MSC were washed and cultured for an additional 24 h in serum-free RPMI with glucose 2 or 4.5 g/L, and the medium was collected (conditioned medium, CM).

### In vitro T-cell proliferation assay

MSC CM equivalent to that of 2 × 10^4^ MSC was assessed for its effect on proliferation of 2 × 10^5^ anti-CD3/CD28-activated spleen cells (1:10 ratio) labeled with CFSE (1 µM; Molecular Probes). Equivalent amount of CM from 3T3 fibroblast culture was used as control. Where the CM of MSC was collected in RPMI with different concentrations of glucose (2 or 4.5 g/L), the final glucose concentration in the T-cell activation medium was 4.5 g/L. Alternatively, MSC CTRL-KD and SLPI-KD were irradiated (5000 rad) and added to 2 × 10^5 anti-CD3/CD28-activated spleen cells (1:10 ratio) labeled with CFSE (1 µM) at the ratio of 1:40. After 72 h, splenocytes were collected and the mean fluorescence intensity of CD3-positive T cells was evaluated by flow cytometry. Statistical differences were evaluated using Student’s *T*-test.

### Real-time PCR

Total RNA was extracted with Trizol (Invitrogen) from MSC, MSC RAPA, MSC CUC, MSC CTRL-KD and STAT1-KD 24 h after treatment with IFNβ. Real-time PCR experiments were conducted as per the manufacturer’s instructions (SYBR Green I Master, Roche) using the following primers:

*Cd274* (aaatcgtggtccccaagc/tcctcatgttttgggaactatct)

*Ccl2* (catccacgtgttggctca/gatcatcttgctggtgaatgagt)

*Hgf* (caccccttgggagtattgtg/gggacatcagtctcattcacag)

*Il18bp* (agctattcggggcttaggag/tgcaagcaagtctggtgtct)

*Slpi* (cttgctctggggatcctg/ggctccgattttgatagcat)

Gene expression values were calculated as 2^(−ΔCT)^ with hypoxanthine guanine phosphoribosyl transferase (*Hprt*) (tcctcctcagaccgctttt/cctggttcatcatcgctaatc) as reference gene. Statistical differences were evaluated using Student’s *T*-test.

### Western blot

Total cell lysates and western blot analysis were performed as previously described^21^. The blots were probed with antibodies specific for pSTAT1 (Tyr701), pSTAT3 (Tyr705), pS6 (all 1:1000 dilution and from Cell Signaling Technology, Beverly, MA) and with an actin antibody (1:1000 from Santa Cruz) to normalize for the amount of loaded protein.

### Pathway inhibition by pharmacological agents

STAT3 inhibition was obtained by exposure of MSC to cucurbitacin I (CUC) (500 nM; Sigma Aldrich) for 3 h in serum-free RPMI; mTOR inhibition was obtained through exposure of MSC to rapamycin (100 nM; Sigma Aldrich) in serum-free RPMI for 1 h (RAPA). The inhibition of STAT3 and S6 phosphorylation following treatments was evaluated by Western blot analysis using phospho-specific antibodies (data not shown).

### Immunofluorescence

MSC were fixed in 4% paraformaldehyde, permeabilized with 0.5% Triton and labeled with antibodies specific for mouse SLPI (1:100 dilution from Cell Signaling) followed by a secondary antibody conjugated with AlexaFluor-594. Nuclei were stained with DAPI. The slides were analyzed with a Olympus microscopy equipped with 405 and 561 nm filters.

### Analysis of MSC metabolism

Real-time measurements of extracellular acidification rate (ECAR) and oxygen consumption rate (OCR) were measured using an XFe-96 Extracellular Flux Analyzer (Seahorse Bioscience). Cells were counted using an automated Cell counter (Countess from Life Technologies), seeded in XFe-96 plates (Seahorse Bioscience) at the density of 2 × 10^4^ cells/well and incubated overnight at 37 °C in 5% CO_2_ atmosphere in the presence or absence of IFNβ (1500UI/ml). ECAR was measured in XF media in basal condition and in response to 10 mM glucose, 4 μM oligomycin and 100 mM of 2-Deoxy-d-glucose (2-DG). Basal glycolysis was calculated after glucose injection (subtracting the ECAR rate inhibited by 2DG). Maximal glycolysis was measured after oligomycin injection and glycolytic capacity as the difference of oligomycin-induced ECAR and 2-DG-induced ECAR. OCR was measured in XF media (non-buffered DMEM medium, containing 10 mM glucose, 2 mM L-glutamine, and 1 mM sodium pyruvate), under basal conditions and in response to 5 μM oligomycin, 1.5 μM of carbonylcyanide-4-(trifluoromethoxy)-phenylhydrazone (FCCP) and 1 μM of antimycin A and rotenone (all chemicals from Sigma Aldrich). Basal OCR was calculated as the difference between baseline measurements and antimycin A/rotenone-induced OCR; maximal OCR was measured as the difference between FCCP-induced OCR and antimycin A/rotenone-induced OCR. Finally, the ATP-linked parameter was calculated as the difference between baseline measurements, without injection, and oligomycin-induced OCR. Experiments with the Seahorse system were done with the following assay conditions: 3 min mixture; 3 min wait; and 3 min measurement. Then, metabolic parameters were calculated. Data are expressed as mean and S.E.M. from four independent experiments. Statistical differences were evaluated using the Wilcoxon matched-pairs test.

## Results

### IFNβ promoted MSC immunomodulation inducing the expression of secreted mediators

**Fig. 1:**
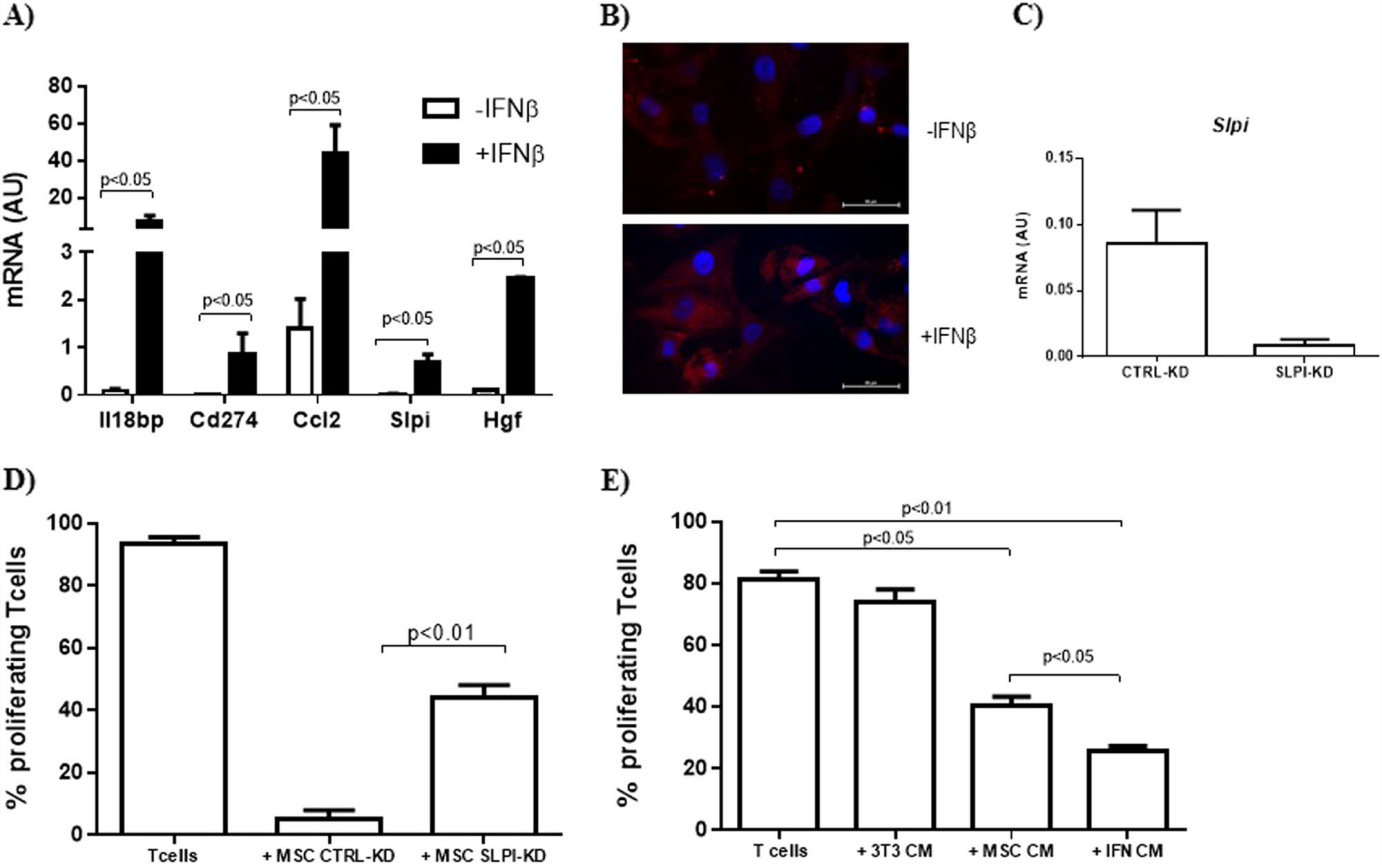
IFNβ induced the expression of soluble mediators and promoted MSC immunomodulatory function. **a** Real-time PCR analysis of immunoregulatory genes in MSC exposed or not to IFNβ. Data are mean ± SD (*n* = 3); **b** Immunofluorescence analysis of Slpi expression in MSC. Red, SLPI; blue, DAPI; **c** Real-time PCR analysis of *Slpi* expression was evaluated in MSC following transfection with specific siRNA (MSC SLPI-KD) and compared to that of control MSC (MSC CTRL-KD). Data are mean ± SD (*n* = 3); **d** In vitro T-cell proliferation assay in the presence of MSC CTRL-KD and MSC SLPI-KD. MSC were added at the ratio 1:40 with activated splenocytes. Data are presented as mean ± SD (*n* = 3); **e** In vitro T-cell proliferation in the presence of MSC conditioned medium (MSC CM) collected after priming with IFNβ (IFN CM). The CM of 3T3 fibroblast culture was used as control. Data are mean ± SD (*n* = 3)

To verify the possibility that IFNβ may affect the expression of immunomodulatory factors secreted by MSC we analyzed, 24 h upon priming with IFNβ, the mRNA levels of *Cd274*, *Ccl2*, and *Hgf*, which are known to act in a paracrine way on immune cells. We also assessed the gene expression of *Il18bp* and *Slpi*, that we recently identified as induced in MSC upon interaction with activated T cells^11^. As shown Fig. 1a, IFNβ induced a significant increase of mRNA level of all the genes analyzed. We then focused on *Slpi*, whose role in MSC immunomodulation has never been reported. We first confirmed that MSC exposed to IFNβ increased Slpi expression at protein level (Fig. 1b). To get some insight into the role of Slpi on MSC immunoregulatory function, we reduced its expression in MSC through gene silencing (SLPI-KD) and used these Slpi-silenced cells to inhibit T-cell proliferation (Fig. 1c). As shown in Fig. 1d, MSC SLPI-KD showed a reduced ability to inhibit T-cell proliferation in vitro as compared to MSC transfected with control siRNA (CTRL-KD).

Then we sought to verify if IFNβ was able to affect the ability of MSC to control T-cell proliferation priming MSC with IFNβ and testing the effect of the derived conditioned medium (CM) on activated T cells. As shown in Fig. 1e, IFNβ significantly enhanced the constitutive immunomodulatory effect of MSC CM.

These results demonstrated that IFNβ induced in MSC the expression of soluble immunomodulatory factors and concomitantly improved their ability to inhibit T-cell proliferation in vitro.

### IFNβ induced dynamic changes in STAT1, STAT3, and S6 phosphorylation

**Fig. 2:**
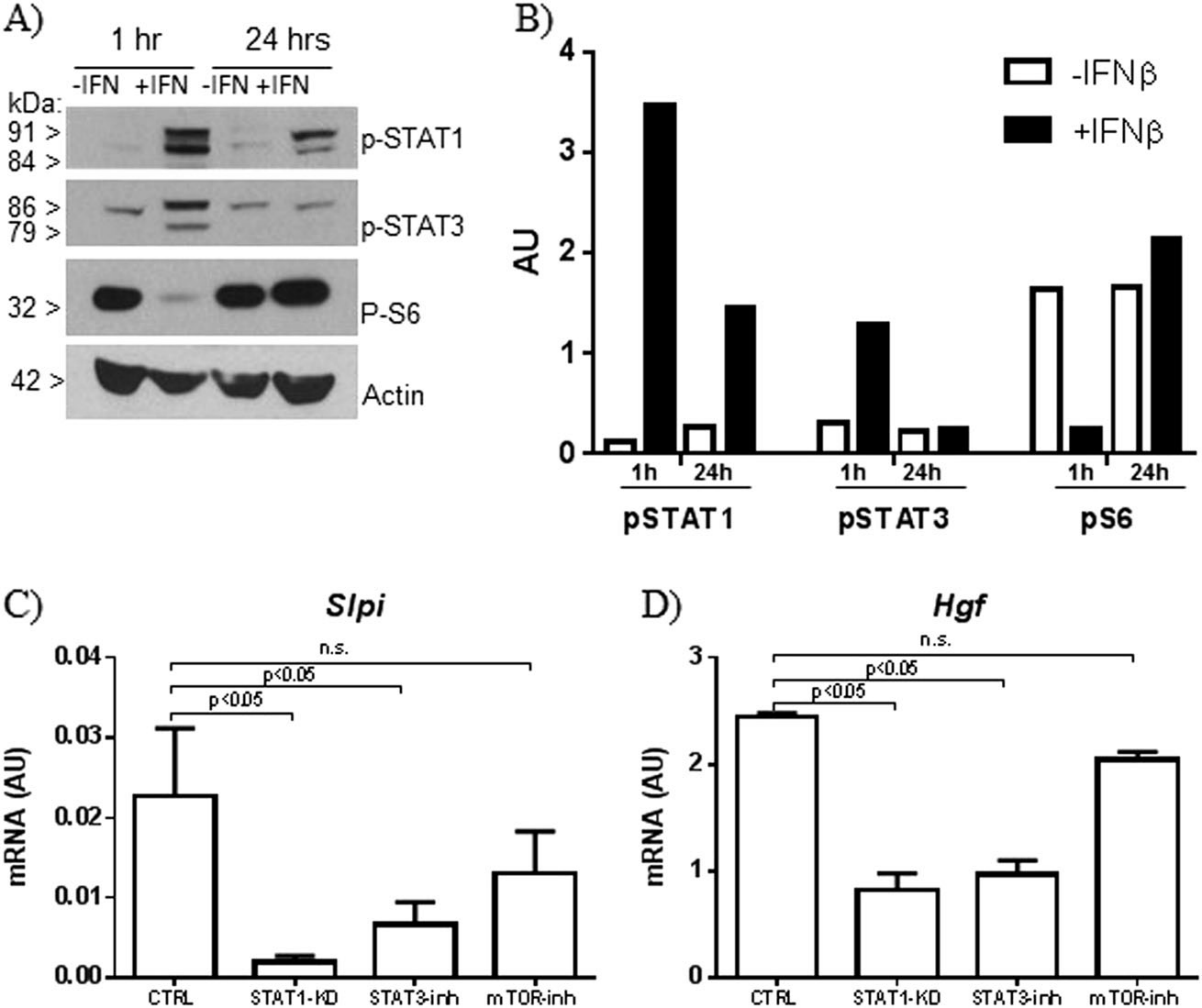
IFNβ induced dynamic changes in STAT1, STAT3, and S6 phosphorylation. **a** Western blot analysis conducted after 1 or 24 h of incubation with IFNβ. Actin served as loading control; **b** relative densitometric quantitation of the shown gel, after normalization on actin; **c**, **d** Real-time PCR analysis of *Slpi* and *Hgf* expression in MSC exposed to IFNβ upon pathway inhibition. Data are presented as mean ± SD (*n* = 3)

As activation of STAT1 and STAT3, and inhibition of mTOR pathway positively affects MSC immunomodulatory function upon administration of interferon gamma (IFNγ)^11^, we analyzed the activation state of these pathways following exposure to IFNβ. Analysis of phosphorylated pathway components revealed that exposure to IFNβ for 1 h dramatically increased the levels of phosphorylated STAT1 (pSTAT1) and STAT3 (pSTAT3) and concomitantly reduced the amount of phosphorylated S6 (pS6), used as read-out of mTOR activity (Fig. 2b). Upon a prolonged exposure (24 h), pSTAT1 and pSTAT3 decreased as compared to 1 h, indicating that IFNβ induced a a transient phosphorylation of STAT1 and STAT3. Concomitantly, the levels of pS6 strongly increased as compared to that observed upon exposure to IFNβ for 1 h, revealing that IFNβ induced an oscillatory modification of mTOR pathway activity (Fig. 2a, b).

### IFNβ-dependent expression of *Slpi* and *Hgf* was mediated by STAT1 and STAT3

Engagment of STAT1 and STAT3 induces the expression of immunomodulatory genes in MSC, that is further enhanced by inhibition of mTOR signaling^11^. To assess the role of these pathways in the induction of *Slpi* and *Hgf* by IFNβ, we inhibited STAT1 through gene silencing, STAT3 using the chemical inhibitor Cucurbitacin I (CUC) and mTOR with its inhibitor rapamycin (RAPA) before the exposure to IFNβ and we analyzed *Slpi* and *Hgf* expression by real-time PCR. We observed that the induction of *Slpi* expression by IFNβ was significantly impaired by STAT1 and STAT3 inhibition, while the blockade of mTOR pathway did not exert any affect on their expression (Fig. 2c). Similarly, we observed that impairment of STAT1 and STAT3 signaling, but not that of mTOR, reduced the induction of *Hgf* upon exposure to IFNβ (Fig. 2d).

### Exposure to IFNβ modified MSC energetic metabolism

**Fig. 3:**
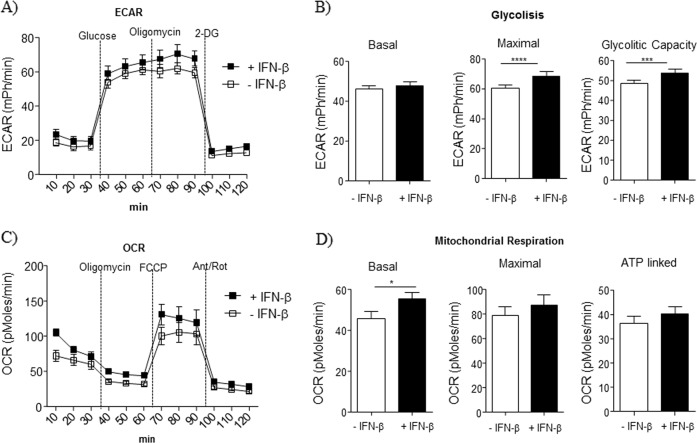
Exposure to IFNβ modified MSC energetic metabolism. **a** Kinetic profile of ECAR in MSC treated or not with IFNβ for 12 h. The data are shown as mean ± S.E.M. of four independent experiments. ECAR was measured in real time, under basal conditions and in response to glucose, oligomycin and 2-DG; **b** parameters of glycolysis in MSC were calculated as detailed in Materials and methods. Data are expressed as mean ± S.E.M. of three measurements, from four independent experiments; **c** kinetic profile of OCR in MSC treated or not with IFNβ for 12 h. The data are shown as mean ± S.E.M. of four independent experiments. OCR was measured in real time, under basal conditions and in response to oligomycin, FCCP and Antimycin A + Rotenone. **d** Parameters of mitochondrial respiration in MSC were calculated as detailed in material and methods. Data are expressed as mean ± S.E.M. of three measurements, from four independent experiments

As IFNβ affected the activity of mTOR pathway, a master regulator of intracellular metabolism, we assessed whether exposure to IFNβ could impact on the bioenergetic profile of MSC, by measuring extracellular acidification rate (ECAR) as an indicator of glycolysis, and oxygen consumption rate (OCR) indicator of mitochondrial respiration. We observed that IFNβ did not modify basal glycolysis in MSC (Fig. 3a, b). However, maximal glycolysis (induced by oligomycin injection) and glycolytic capacity were significantly increased by IFNβ treatment, as compared to untreated MSC (Fig. 3a, b). Then we investigated mitochondrial respiration in terms of OCR. IFNβ did not affect maximal OCR and OCR coupled to ATP generation, but slightly enhanced the respiratory capacity of MSC in basal conditions (Fig. 3c, d), indicating that IFNβ preferentially increased the glycolytic capacity of MSC.

### Glycolisis engagement improved MSC immunomodulatory function

**Fig. 4:**
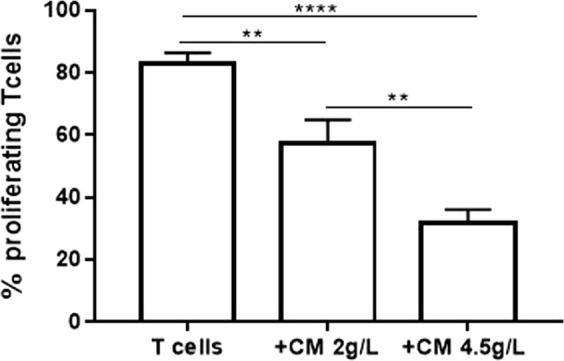
Promotion of MSC glycolisis improved their ability to inhibit T-cell proliferation in vitro. In vitro T-cell proliferation assay in the presence of conditioned medium of MSC (MSC CM) collected in serum-free RPMI at different concentration of glucose (2 g/L or 4, 5 g/L). The final glucose concentration in the T-cell activation medium was 4.5 g/L. Data are mean ± SD (*n* = 3)

To link MSC glucose metabolism with their immunomodulatory function, we assessed whether or not improvement of MSC glycolitic rate may impact on their capacity to inhibit T-cell proliferation. We boosted glycolitic flux by growing MSC in high glucose containing medium (4.5 g/L), and we assessed the immunomodulatory properties of the resulting CM in T-cell proliferation assay. We obtained a significant improvement in the constitutive ability of MSC CM to inhibit T-cell proliferation by collecting MSC CM in high glucose-containing medium as compared to MSC CM prepared in normal medium (2 g/L) (Fig. 4).

These results clearly demonstrated that promoting glucose metabolism improved the immunomodulatory function of MSC, thus suggesting a new possible mechanism through which IFNβ enhances MSC immunomodulation.

## Discussion

While the potent immunomodulatory effect of IFNβ on cell types of the immune system has been extensively studied^22^, little is known on the interaction between IFNβ and MSC. IFNβ was shown to influence the expression of chemokines and their receptors in human MSC^23^, but the effects of IFNβ on MSC immunomodulatory function are largely unknown. Our data demonstrated that IFNβ significantly increased the expression of molecules, secreted by MSC, with reported immunomodulatory activities. Among those, we observed that Slpi, a molecule never related to MSC biology, potentiated the ability of MSC to control T-cell proliferation in vitro.

Slpi is a polypeptide secreted by epithelial cells to protect tissue from damage by leucocyte proteolytic enzymes. Nevertheless, the regulatory function of Slpi on adaptive immunity is independent to its anti-protease activity. Slpi has been demonstrated to affect the monocyte pattern secretion by preventing the interaction of p65 with the NF-kB binding sites^24^, to inhibit effector T-cell proliferation^25^ and to promote the generation of regulatory T cells^26^. Slpi is upregulated in macrophages, activated microglia, neuronal cells and astrocytes during EAE attack^27^. Interestingly, Slpi was shown to increase proliferation of adult neural stem cells promoting oligodendroglial differentiation, as incubation of adult neural stem cells with recombinant Slpi resulted in an increase of cell proliferation and of differentiation towards oligodendrocytes^27^. Whether Slpi secreted by MSC may play a role also in tissue repair promoted by MSC requires further investigation. Interestingly IFNβ significantly enhanced also the expression of Hgf a pleiotropic cytokine with neuroprotective which has been demonstrated to enhance axonal outgrowth and oligodendroglial maturation^12^.

At molecular level, IFNβ induced a transient phosphorylation of STAT1 and STAT3, determining the induction of *Slpi* and *Hgf* expression, that was indeed impaired by both STAT1 and STAT3 inhibition. Concomitantly, IFNβ induced a dynamic modification of mTOR activity, that was early reduced and then increased. In line with the role of mTOR in the control of glucose metabolism, a prolonged exposure to IFNβ increased MSC glycolytic capacity, thus improving their ability to engage glycolysis to convert glucose to pyruvate or lactate. Energy metabolism and functional activation are fully integrated in immune cells to determine their ability to divide, differentiate, and carry out effector functions^28^. Here we found that inhibition of T-cell proliferation by MSC was strongly enhanced by boosting MSC glycolisis. As ECAR, monitored as readout of MSC glycolitic activity, was mainly due to production of lactate, our data suggest the possible involvement of lactic acid in MSC immunomodulatory function. Lactic acid had been shown to suppress the proliferation and cytokine production of human cytotoxic T lymphocytes^29^ and to inhibit TNF secretion and glycolysis of human monocytes^30^.

Our data support that IFNβ can enhance MSC therapeutic function through two different molecular mechanisms: the early induction of STAT1-3-dependent immunomodulatory genes, and the long-lasting mTOR-associated promotion of glucose metabolism. Particularly, based on the finding that IFNβ induced the expression of cytokines with both immunomodulatory and neuroprotective features such as Hgf and Slpi, we speculate that MSC as add-on therapy to IFNβ treatment could promote tissue repair beyond modulation of inflammatory reactions. In line with these results, administration of IFNβ-secreting engineered MSC has been demonstrated to be more effective than wild-type MSC in the attenuation of EAE^31,32^, further supporting the rationale for combined protocols to treat MS. Moreover, the finding that MSC immunomodulatory function is improved by glucose metabolism revealed a new possible mechanism underlying MSC therapeutic effect.
